# Building a green Belt and Road: A systematic review and comparative assessment of the Chinese and English-language literature

**DOI:** 10.1371/journal.pone.0239009

**Published:** 2020-09-15

**Authors:** Hoong Chen Teo, Ahimsa Campos-Arceiz, Binbin V. Li, Mingquan Wu, Alex Mark Lechner

**Affiliations:** 1 School of Environmental and Geographical Sciences, University of Nottingham Malaysia, Semenyih, Selangor, Malaysia; 2 Southeast Asia Biodiversity Research Institute, Chinese Academy of Sciences, Yezin, Nay Pyi Taw, Myanmar; 3 Center for Integrative Conservation, Xishuangbanna Tropical Botanical Garden, Chinese Academy of Sciences, Menglun, Yunnan, China; 4 Environmental Research Center, Duke Kunshan University, Kunshan, Jiangsu, China; 5 Nicholas School of the Environment, Duke University, Durham, NC, United States of America; 6 State Key Laboratory of Remote Sensing Science, Aerospace Information Research Institute, Chinese Academy of Sciences, Beijing, China; 7 Centre for Water and Planetary Health, School of Geography, University of Lincoln, Lincoln, United Kingdom; Institute for Advanced Sustainability Studies, GERMANY

## Abstract

International attention on the environmental impacts of China’s Belt and Road Initiative (BRI) is increasing, but little is known internationally about the large corpus of Chinese BRI environmental research. We present the first systematic review of the Chinese and English-language BRI environmental research, supported with text mining and sentiment analysis. We found that the research is dominated by Chinese authors writing about BRI routes within China in Chinese, even though concerns around BRI are largely about impacts and benefits within host countries, and the volume of publications in English is recently catching up. Different disciplines and methods are well-represented across languages, apart from specific types of Chinese social science papers. The sentiments of academic research are largely neutral and less polarised than media discourse. We recommend that scientists and practitioners should pay more attention to BRI environmental impacts in developing countries and proactively engage local voices.

## Introduction

China's multi-trillion dollar Belt and Road Initiative (BRI) is the largest infrastructure scheme in our lifetime, involving multilateral actors in constructing a network of infrastructure and economic cooperation corridors across Eurasia [1]. It will profoundly reshape the geographies of Asia and beyond, and has also become a lightning rod for anxieties about global geopolitical, economic, and environmental changes that the 21st century will bring [2]. In spite of the increasing interest on BRI by environmental researchers, little is known internationally about the even larger corpus of BRI environmental research conducted in China. In other domains, fears abound of the economic and cyber balkanisation of the world order [3], which are liable to shatter China's dreams of fostering closer connectivity and cooperation with BRI [4]. Also, mainstream discourse about China is often strongly polarised [5]. Does this polarisation similarly extend to environmental research about the BRI? Given the high stakes involved with BRI's massive scale [6, 7], this is a question that needs to be urgently answered.

Since the BRI’s announcement in 2013, research on BRI environmental issues has grown to encompass a plurality of disciplines and approaches. It is thus timely to present the first systematic review of Chinese and English-language BRI environmental research. This can provide a comprehensive picture of international and Chinese BRI research, and highlight possible differences and biases in the two corpuses. Monolingual systematic reviews can be systemically biased, such as when positive and significant results are reported only in English [8, 9]. Other reasons, such as methodology, intended audience, and language barriers may influence authors’ decisions in which language to publish, thus skewing the body of knowledge contained in different languages [10].

Systematic reviews of scientific papers often use methods such as citation metrics and topic modelling which provide objective information [11, 12], but rarely sentiment analysis which aims to extract subjective opinions and attitudes from language [13]. At its most basic form, sentiment analysis indicates polarity (positivity or negativity) and is thus commonly used to capture attitudes of different groups [14, 15]. Sentiment analysis can bolster systematic reviews by identifying attitudes of groups of researchers, which is important for environmental research as there is the potential for scientists to cross the line from science into advocacy for their favoured ecosystem or species [16, 17], and in the case of BRI, for the author’s own political biases. Much of the politically-polarised discourse around BRI can be observed in the media, which can both reflect popular attitudes and shape public opinion [18, 19]. To our knowledge, there are no prior studies comparing sentiment in the media with journal papers.

In this paper, we present a systematic review of BRI environmental research published in both English and Chinese languages. We first examine the geographical characteristics of this research, including who and where the research is coming from, how much international collaboration there is, how it is funded, and the spatial coverage of the research. Secondly, we examine methods and content to characterise what is being researched. Thirdly, we examine how positive environmental researchers are about BRI, using sentiment analysis to compare languages and authors. To provide an independent measure of relative sentiment, we then compare the sentiments expressed in academic BRI environmental research with media coverage, as well as another contested environmental issue, oil palm [20]. Finally, we discuss prospects and implications for future research in this field.

## Materials and methods

**Fig 1:**
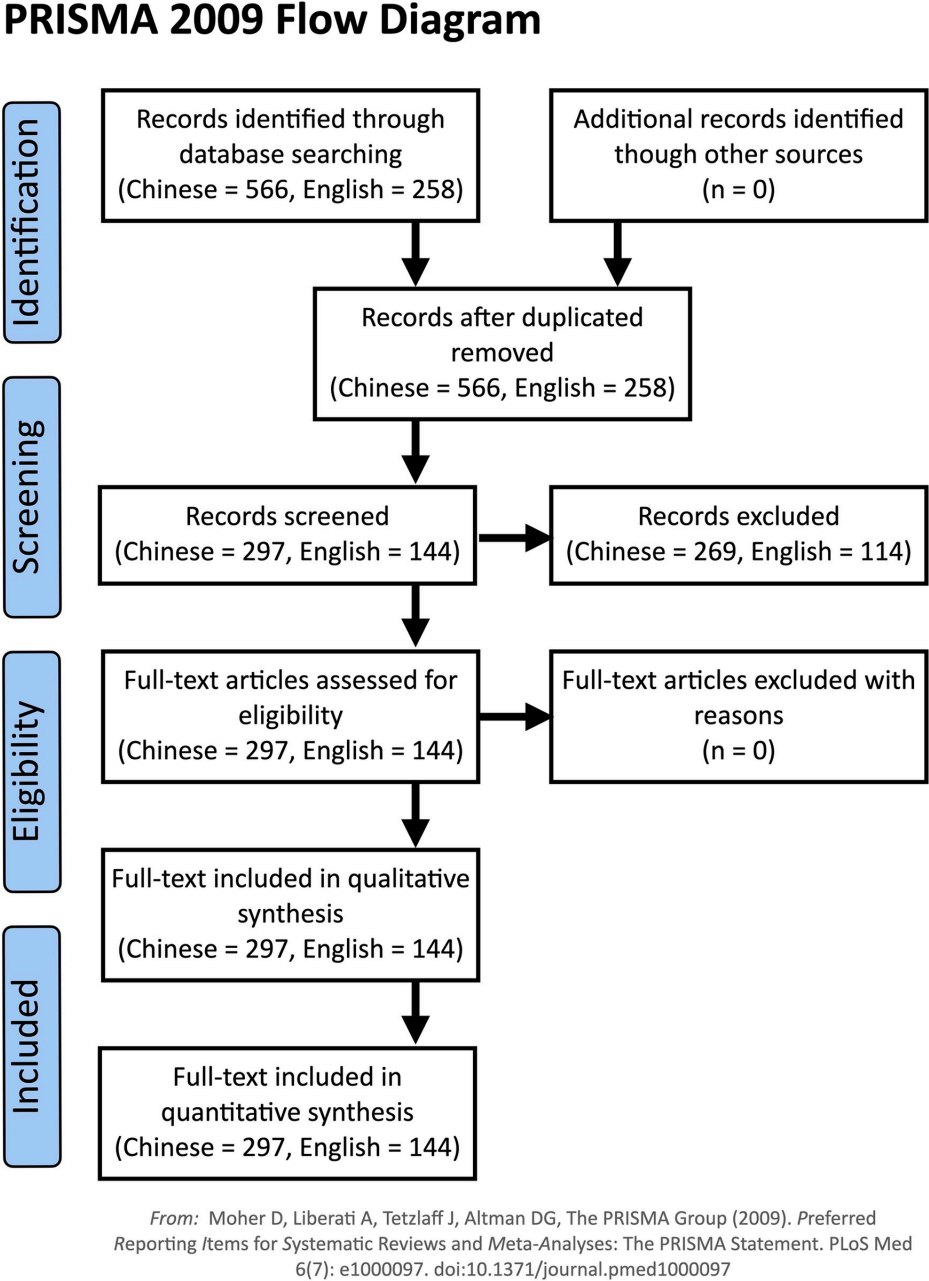
Flow diagram indicating the number of items used for analyses in this study.

A search string capturing a broad range of environmental papers from various disciplines was formulated, consisting of the terms “ecology”, “environment”, “green”, and various permutations of BRI (see Appendix A in S1 Text for full search string). The search string was queried on the China National Knowledge Infrastructure (CNKI) and Scopus databases for the period 1 Jan 2013 to 30 Jun 2019. CNKI is the main academic journal database for China and is frequently used for systematic reviews in a similar way to Scopus for English-language journal papers [21]. The search was applied to the Abstract, Title, and Keywords of journal articles, returning 566 results from CNKI and 258 on Scopus. Manual screening excluded irrelevant articles, such as the numerous papers referring to “investment environment” in Chinese, leaving 297 Chinese-language papers and 144 English-language papers (Fig 1).

First, each paper was coded for its provenance, methods, scope, and content (see Table 1). A list of themes was extracted from “The Belt and Road Ecological and Environmental Cooperation Plan” (hereafter referred to as “Green Plan”; [22]), and each paper was coded according to the most proximate theme.

**Table 1:** List of variables and response categories derived from “The Belt and Road Ecological and Environmental Cooperation Plan” [22].

Variable	Response categories
Methods	Theoretical, Empirical
Type of paper	Commentary, Review, Framework, Original research^1^
Discipline	Economics, Geography, Policy, Law, Finance, Others^2^
Belt and Road Ecological and Environmental Cooperation Plan themes^3^	(1) Rationale, principles and goals (2) Green policy coordination and communication (3) Promote green production and infrastructure through laws and regulations (4) Sustainable production, consumption and trade (5) Green financing (6) Socio-environmental protection schemes (7) Capacity-building and safeguards
Funding	None, NGO, College, Provincial, Government agencies, National, Foreign
Routes	Routes within China, Other routes, All routes^4^
Scale	Site, Subnational^5^, National, International

^1^ Refers to papers collecting or synthesising original material, including econometric and remote sensing papers analysing data.

^2^ Each paper was assigned one discipline. Disciplines were determined based on content of the paper and affiliation of the first author. Papers explicitly addressing financial issues, such as green financing and green bonds, were classified as finance rather than economics.

^3^ Refer to S1 Table in S1 Text.

^4^ Including general research not specific to any route.

^5^ Provincial or regional.

Secondly, text mining was conducted in R 3.5.3. [23] using the ‘tm’ [24] and ‘tmcn’ [25] packages. Chinese words were segmented using ‘Rwordseg’ [26], then translated into English using Google Translate. Machine translation is often the only feasible approach for comparative researchers working across languages, and studies have suggested that using Google Translate to translate text from other languages into English delivers accurate results for topic modelling [27, 28]. The corpus containing both Chinese and English documents was processed with text mining transformations to strip punctuation, numbers, whitespace, and stopwords.

Thirdly, data ordination using Detrended Correspondence Analysis (DCA) was performed on the document term matrix constructed from the corpus using the ‘decorana’ function in the ‘vegan’ package [29] to derive a cluster plot showing the relation between papers.

Finally, sentiment analysis was conducted on the BRI and oil palm environmental papers and news articles to compare the sentiments expressed in academic BRI environmental research with media coverage and another contested environmental issue, oil palm. We used the ‘sentimentr’ package [30], which offers sentence-level sentiment detection using an augmented dictionary-lookup approach taking into account valence shifters. Valence shifters, such as negators or amplifiers, flip or intensify a polarised word, thereby offering more contextual nuance than a dictionary lookup tool. For the media coverage assessment, 141 English-language and 210 Chinese-language news articles on BRI and the environment between 1 May 2019 and 30 Jun 2019 were downloaded from the Factiva database. Both English and Chinese-language news articles included sources from Mainland China and overseas; most of the English-language news articles came from international sources and most of the Chinese-language news articles were from Mainland China. For the assessment of oil palm sentiment, 386 oil palm environmental papers between 2014 and 2019 were downloaded from Scopus, as well as 239 news articles between 1 Jan 2019 and 30 Jun 2019 from Factiva. All analyses were performed in R version 3.5.3. [23].

## Results

### Overview

**Fig 2:**
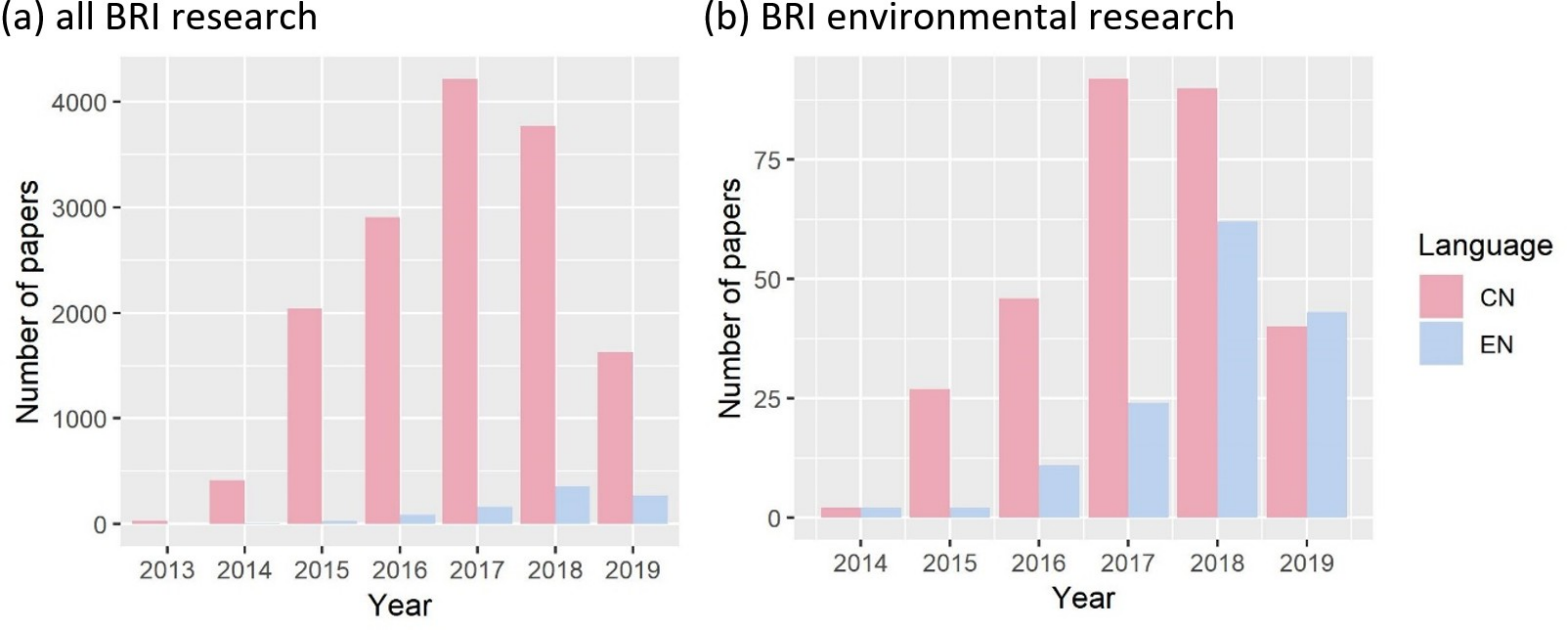
Number of papers in CNKI and Scopus databases on **(a)** all BRI papers, and **(b)** BRI environmental issues. *NB*. *2019 data only for the first six months*.

Research on BRI increased rapidly since 2013, but appears to be tapering off from 2018, especially for Chinese-language papers (Fig 2). Searching in the China National Knowledge Infrastructure (CNKI) and Scopus academic databases we identified a total of 15,930 papers on the BRI, of which only 441 pertained to the environment. As such, papers on environmental research only formed a small proportion (2.8%) of the overall research on BRI regardless of language or year. Furthermore, the volume of papers on BRI environmental research appears to lag behind general BRI research and does not appear to be tapering off. English-language papers formed a larger proportion (33%) of BRI environmental research compared to all BRI research (5.8%). The rest of the manuscript focuses only on the 441 BRI environmental research papers.

### Authors

**Fig 3:**
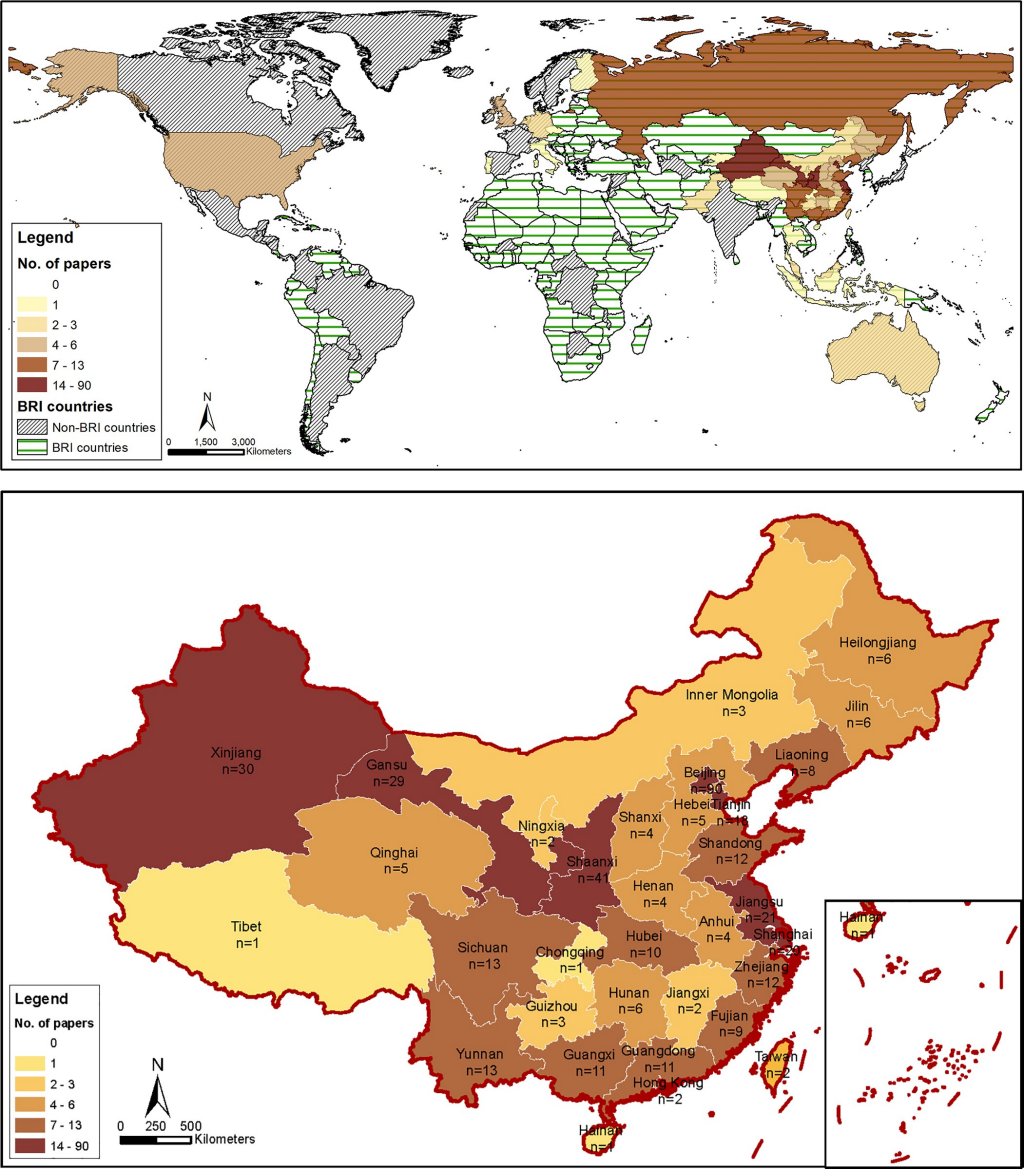
**(a)** First author institutional affiliation of all Chinese- and English-language BRI environmental papers. **(b)** First author institutional affiliation of all Chinese- and English-language BRI environmental papers published in China. N.B. ‘BRI countries’ are those which have signed BRI cooperation documents with China, *ca* January 2020 [32].

Most (92%) of the first authors of both Chinese and English BRI environmental papers were based in Chinese institutions (Fig 3A). Out of 144 English-language papers, only 12% were led by first authors affiliated to an institution in the US, Europe, and Australia; and only 12% were led by a first author in another BRI country. All 297 Chinese papers were published by first authors in China, including authors from every province, across first, second, and third tier cities [31]. Chinese authors from diverse affiliations were represented, including the Chinese Academy of Sciences, Communist Party Schools, large research universities, small provincial colleges, government-linked corporations, and private corporations (S2 Table in S1 Text). Within China, most of the first authors were from the traditional Silk Road provinces of Xinjiang, Gansu, and Shaanxi as well as the main academic and political centres of Beijing, Tianjin, and the Yangtze River Delta (Fig 3B).

Most of the BRI environmental papers did not involve international collaborations. None of the Chinese-language papers had foreign-based co-authors; while 56% of English-language papers had authors based in China only, 18% were written by foreign-based authors in the same country, and 2% were collaborations between foreign-based co-authors in more than one country. Only 24% (35 papers) of English-language papers involved collaboration between authors based in China and overseas. 18 of those papers were collaborations only between co-authors in Western countries (Europe (excluding Russia), US, Canada, Australia and New Zealand), while 17 involved at least one co-author in a non-Western country.

### Funders

**Fig 4:**
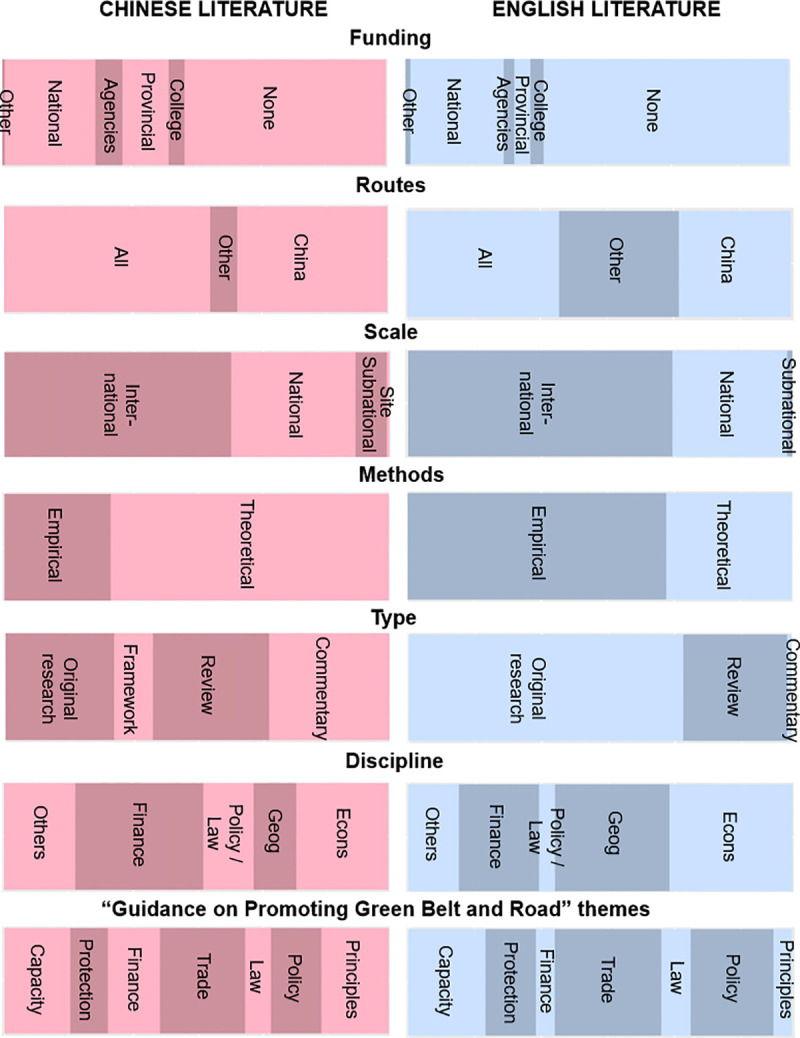
Proportions of papers on a scale of 0–100% according to provenance, methods and content for Chinese- and English-language BRI environmental papers.

A larger proportion of the Chinese literature (47%) was funded; compared to the English literature (36%). All funded papers, except four, received funding from Chinese state-related entities of different levels–national research funds, state agencies, provinces, cities and universities (Fig 4). The proportion of funded Chinese papers has been largely consistent throughout the years, but all except two of the funded English papers were published from 2018 onwards.

### Geographical focus

Chinese portions of the route were addressed by a large proportion of papers in the Chinese corpus (39%) and English corpus (29%). Only 7% of Chinese papers were specific to a BRI route outside China, while 31% of English papers studied a BRI route outside China (Fig 4).

### Methods and content

A higher proportion of English papers (67%) used empirical methods than the Chinese literature (28%). There was also a stronger focus on the principles, rationale, and goals for a green BRI in the Chinese literature. Also, English-language papers displayed a lack of engagement with Chinese key BRI environmental policy documents, i.e. “The Belt and Road Ecological and Environmental Cooperation Plan” [22] and “Guidance on Promoting Green Belt and Road” [33]. Only 5 (3.5%) English-language papers referred to either document, compared to 63 (21%) Chinese-language papers mentioning either one or both. A range of disciplines and themes were generally well-represented in both English and Chinese literature. Overall, as suggested by the top 10 most frequently occurring terms in all papers, environmental issues were frequently discussed from environmental, geographical, and economic perspectives (Table 2).

**Table 2:** Top 10 terms and frequency of occurrence in all papers. During analysis, all terms were stemmed.

Term	Frequency
development	20,766
environment	19,812
green	12,901
economy	11,415
ecology	10,113
region	8,160
industries	7,872
resource	6,983
energy	6,339
construction	6,227

### Disciplines

**Fig 5:**
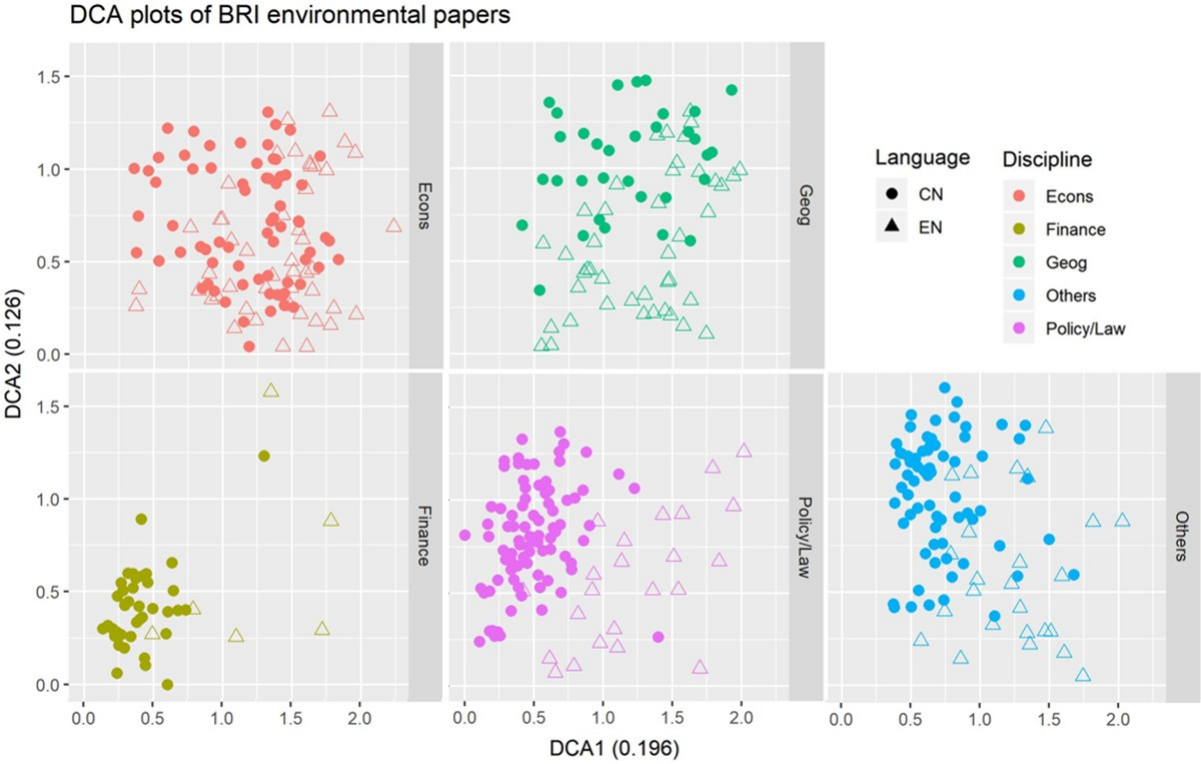
BRI environmental papers were text mined to generate a document term matrix, which was then reduced by detrended correspondence analysis (DCA). The DCA plots show the second detrended correspondence axis (DCA2) against the first detrended correspondence axis (DCA1). Point shape reflects language of the paper and colour reflects the discipline.

The body of literature analysed encompassed different disciplines and themes (Figs 3 and 4). Generally, there was considerable overlap in the terms used by papers from different disciplines, although there was a large body of Chinese-language finance, policy, and law papers with little overlap with English-language papers (Fig 5) and thus less visible to international researchers. As these were mostly qualitative social science papers, there may be greater sociocultural and linguistic barriers between Chinese and international research compared to quantitative physical science papers.

### Sentiment analysis by discipline, author country, funding, and language

**Fig 6:**
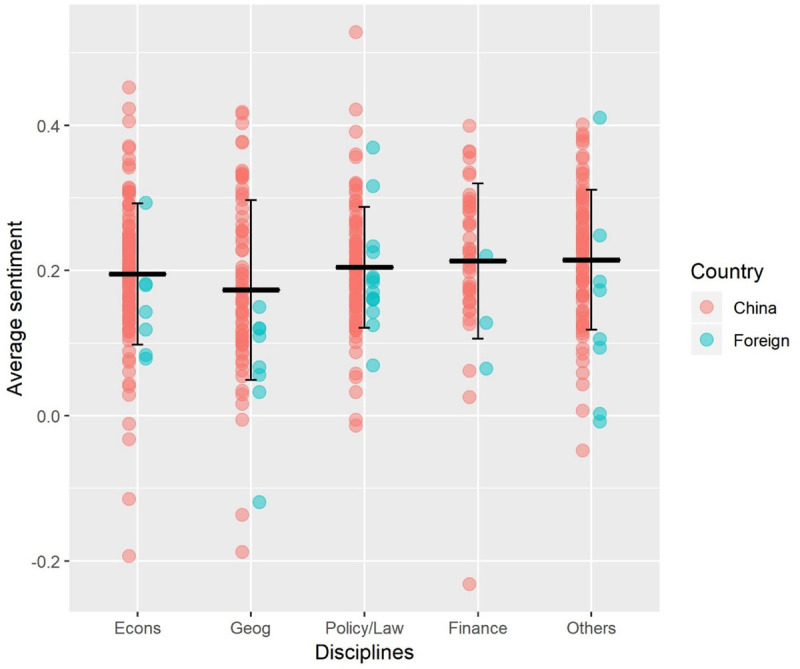
Sentiment analysis results by country of affiliation (point colour) and discipline (x-axis). Horizontal line indicates mean for each discipline and error bars indicate standard deviation of document averages. Note that sentiment analysis was run on each sentence, so each document has a mean and standard deviation. Standard deviation is then derived for each discipline from the variance of document averages.

**Fig 7:**
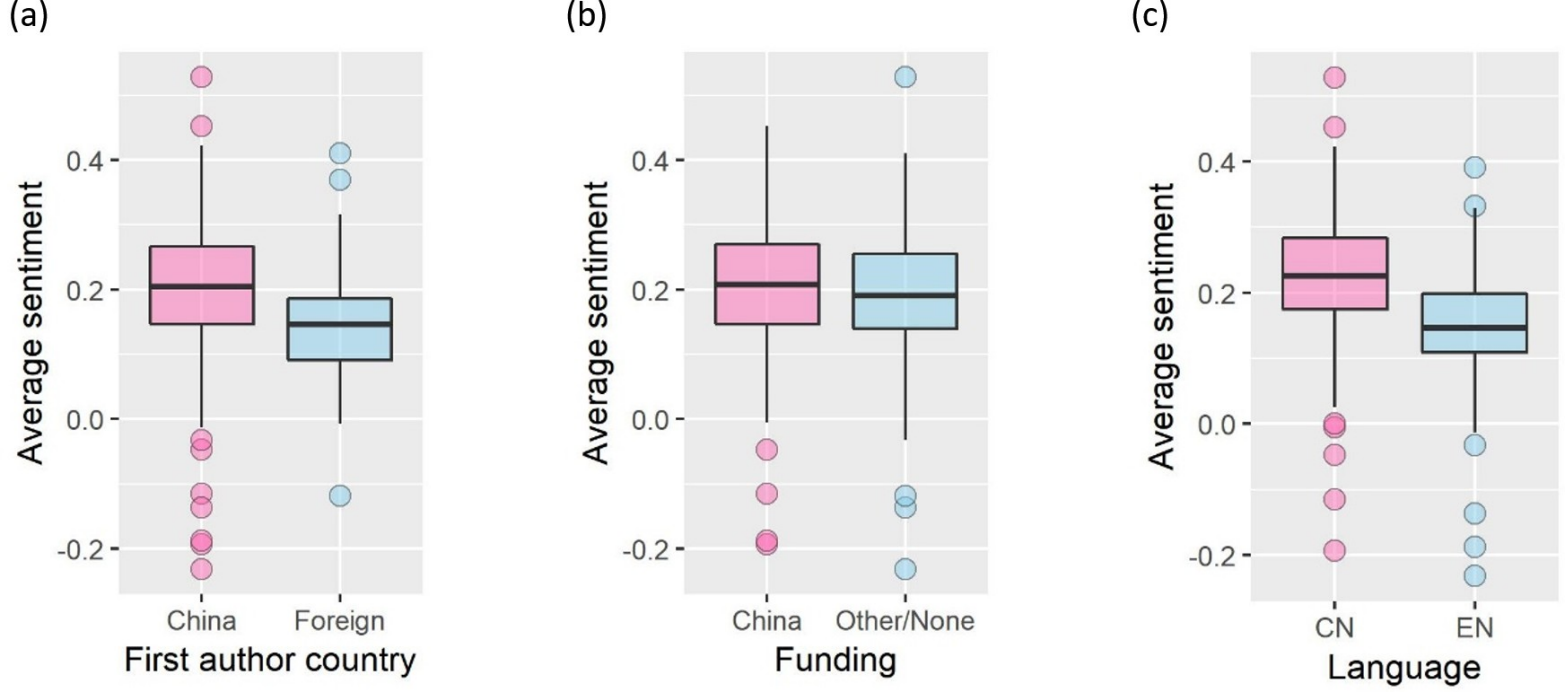
Comparison of average sentiment for **(a)** Chinese and foreign first authors, **(b)** China-funded papers and papers not funded by China, and **(c)** Chinese- (CN) and English-language (EN) papers written by China-based first authors. Horizontal line in boxplot indicates median.

Sentiment analysis revealed that most papers expressed modest positive sentiments (overall mean ± SD sentiment = 0.20 ± 0.10, range = -0.23–0.53; Figs 5 and 6). Only 11 (2.7%, n = 401) Chinese authors and 2 (5%, n = 40) foreign authors expressed negative sentiments on average (Fig 6). We found no difference in average sentiment between disciplines (one-way ANOVA: *F*_(4,317)_ = 1.1, *p* = 0.36). We then tested for differences in average sentiment between author countries, funding sources, and languages (among China-based authors) using independent two-sample t-tests. First authors with a Chinese affiliation were more positive (mean ± SD sentiment = 0.20 ± 0.091, n = 401) than authors with a foreign affiliation (0.15 ± 0.098, n = 40); t(47) = 3.4, *p* < 0.01, power = 0.93 (Fig 7A); there was no difference in sentiment between China-funded papers (0.21 ± 0.10, n = 189) and all other papers (0.19 ± 0.099, n = 252); t(399) = 1.2, *p* = 0.22, power = 0.22 (Fig 7B); and China-based first authors were significantly more positive when writing in Chinese (0.23 ± 0.090, n = 296) than English (0.14 ± 0.099, n = 105); t(169) = 7.4, *p* < 0.01, power = 1 (Fig 7C). Negative sentiments, however, were not necessarily indicative of criticism about the BRI but included concerns about environmental problems in both China and host countries. Samples of text expressing positive and negative sentiments are available in Appendix B in S1 Text.

### Sentiment analysis comparing academic research with coverage in mass media

**Fig 8:**
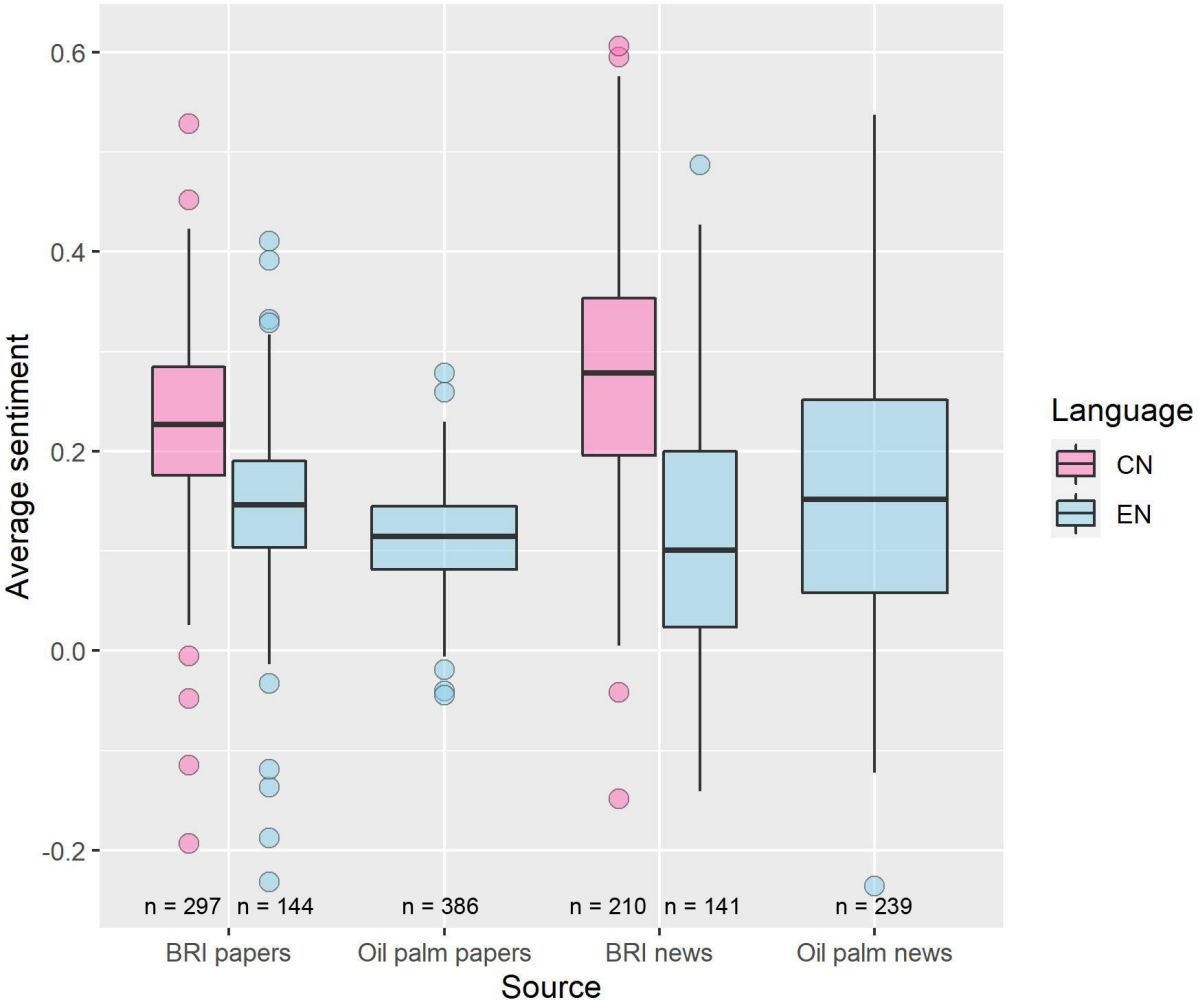
Comparison of average sentiment for BRI and oil palm environmental papers and news articles. Horizontal line in boxplot indicates median.

We found differences in sentiment between BRI environmental academic papers (Chinese, mean ± SD sentiment = 0.23 ± 0.09, n = 401; English, 0.14 ± 0.10, n = 40), BRI environmental news articles (Chinese, 0.28 ± 0.13, n = 210; English, 0.12 ± 0.13, n = 141), oil palm environmental academic papers (English; 0.11 ± 0.05, n = 386), and oil palm news articles (English; 0.13 ± 0.10, n = 239; one-way ANOVA: *F*_(5,1411)_ = 101, *p* < 0.01, power = 1; Fig 8). Post-hoc Tukey HSD tests showed that most (10 out of 15) pairs were significantly different (*p* ≤ 0.015); the five non-significantly different pairs were oil palm news against oil palm papers and BRI English news (*p* = 0.21, 0.89), as well as BRI English news against BRI English papers, oil palm papers and oil palm news (*p* = 0.42, 0.97, 0.91; Fig 8). From these results, environmental news articles showed more polarisation than environmental academic papers, with news articles having a higher total range and interquartile range than papers, and larger differences between English-language and Chinese-language texts for BRI environmental news articles compared to BRI environmental papers. For both BRI environmental news and BRI environmental papers, English-language texts were generally more negative than Chinese-language texts. Also, academic papers on oil palm environmental impacts were less positive than BRI environmental papers in either language (Fig 8).

## Discussion

Most BRI environmental research appears to originate from centres of academic and political power in the West and East rather than on the frontline of where the BRI impacts are. Internationally, the predominance of Chinese authors in BRI environmental research is expected, given that BRI is a Chinese-led project. Much of the non-Chinese research comes from developed Western nations, with better research capacities [34] and interest in international development and large-scale environmental processes, as well as potentially in countering China’s rise. Most of these Western countries do not host BRI projects. Interestingly, the amount of research published in English appears to be overtaking research in Chinese, which may reflect this growing concern and awareness of BRI.

The predominance of literature in Chinese, written by Chinese authors from a Chinese perspective, indicates that much of the literature may be targeted for domestic consumption and thus may not contribute to the international discourse on BRI or address issues of concern for BRI host countries. Similarly, our analyses might be missing BRI-related environmental research conducted in other countries and published in their local languages.

The very low representation of first and co-authors from developing countries on the frontline of BRI investment is concerning; for example, co-authors from China’s immediate neighbours Myanmar, Laos, and Vietnam, which harbour the important Southeast Asian biodiversity hotspots [35, 36] are not represented. Within China, BRI-related research is dominated by the influential urban centres and traditional Silk Road provinces. Chinese provinces outside the traditional Silk Road, such as Yunnan and Guangxi, are directly involved in numerous BRI connectivity projects with neighbouring countries yet less research is coming from them. These also tend to be less developed provinces. The existing research is heavily skewed towards portions of the routes within China, rather than internationally. Possible reasons could be that BRI projects within China were initiated earlier than overseas investments, but also limited data and engagement with BRI host countries, or a lower level of awareness or interest within China of BRI’s impact on these countries. China’s efforts to promote ‘big data’ information-sharing platforms for BRI [37] will hopefully alleviate this and promote new opportunities for academic exchange and research [38]. Meanwhile, the dearth of environmental research specific to BRI routes outside of China should be addressed by the scientific community.

Across the developing world, China is undertaking BRI projects in areas where there is commonly a lack of capacity, expertise, and funding for environmental and social impact assessments [36]. Supporting research by institutes in BRI host countries and provinces can ensure the research is embedded in the local context, utilises local knowledge, has stakeholder buy-in, and demonstrates transparency. It is also important that any funding be provided without expectation about the direction of the research findings. Outside researchers should proactively engage collaborators from BRI countries and provinces. Also, collaboration between Chinese and non-Chinese authors in BRI countries on manuscripts written in Chinese can better ensure that perspectives from overseas are understood in China.

The range of disciplines, themes, topics, and methods in BRI-related research indicates that multiple social and environmental dimensions of BRI are being addressed. Spatial approaches are often used, with GIS and remote sensing techniques being applied to disciplines such as spatial econometrics [39, 40] and landscape ecology [41, 42]. Economic studies on the environment conceptualise environmental processes and systems as exchange value, such as ecosystem services [43]; they also use quantitative environmental variables, most commonly emissions, as a proxy for environmental impacts, and compare them with economic variables such as green total factor productivity [44]. Finally, many policy, law, and finance papers approach environmental management through the lens of social relations between people and groups. However, there may be a gap in understanding between international and Chinese social science research, possibly due to sociocultural and linguistic differences, as our analyses (e.g., DCA cluster plot in Fig 4) suggest. Our description of paradigms and approaches used can hopefully aid researchers in identifying avenues for interdisciplinary research, generating more integrated understandings of the wide-ranging multi-scale nature of BRI environmental impacts [6, 45]; also, especially in qualitative and social science topics, researchers should be mindful of sociocultural differences and seek to understand other perspectives.

Our sentiment analyses show that Chinese authors were more positive than foreign authors, Chinese-language papers were more positive than English-language papers, and China-based authors were more positive when writing in Chinese than English. However, China-funded papers were not more positive than papers not funded by China, suggesting that funding did not sway author sentiment. It should be noted that sentiment analysis captures attitude but not content of the criticism, which could be directed at projects, governments, or the environmental impacts themselves. However, it is important to note that although differences in sentiment were significant, the magnitude of positive sentiment was not high. Nevertheless, BRI environmental papers are far less polarised than news coverage. It is well recognised in both China and abroad that science can provide solutions to ensure the success of BRI [37, 38], and apart from the practical fruits of scientific research, scientific collaboration itself can be a powerful instrument of peace to transcend and alleviate geopolitical and cultural tensions [46].

Oil palm represents a similarly contested environmental issue occurring at a global scale, with stakeholders both in the developed and developing world holding contrasting views. We found that papers on oil palm environmental impacts were less optimistic than both Chinese and English BRI environmental papers. This is partly because a large number of BRI environmental papers do not just highlight impacts but also offer policy recommendations, which are more likely to have a positive tone. However, overall the sentiment values for oil palm were also positive.

## Conclusion

With this systematic review we found that BRI environmental research has addressed a range of social and environmental issues, across different disciplines, topics and methods. These are generally well-represented across both English and Chinese-language corpuses, except for some Chinese social science papers. Our text mining indicated that papers clustered into three main clusters–spatial, economic, and social. Interdisciplinary approaches should aim to bridge the gaps between these clusters. Geographically, research was dominated by Chinese authors writing about BRI routes within China in Chinese, with very few authors from developing countries on the frontline of BRI investment. Sentiments in academic research were largely neutral and less polarised than news articles. This underscores the role of science and academic research as a sensible and rational basis for cooperation, which BRI stakeholders should prioritise. As such, we suggest that BRI projects should include support for research, a pre-condition of which should be the involvement of research partners from BRI host countries and provinces. Chinese funders and authors should also prioritise collaboration with non-Chinese authors, especially on studies published in Chinese and in the social sciences. In this paper, we highlighted potential geographic and interdisciplinary gaps and opportunities for the scientific community to address. Further research can focus on identifying barriers to collaboration and solutions to eliminate these gaps.

## Supporting information

S1 Checklist PRISMA 2009 checklist. (DOC)

S1 Text Supporting information including Appendices A and B and S1 and S2 Tables.
(DOCX)

S1 Dataset List of articles included in the analyses. (XLSX)

S2 Dataset Results of sentiment analysis. (CSV)
