# Supplementary material for: Building a green Belt and Road: A systematic review and comparative assessment of the Chinese and English-language literature
Source: PLoS One. 2020 Sep 15;15(9):e0239009. doi: 10.1371/journal.pone.0239009 (PMC7492099; doi:10.1371/journal.pone.0239009)
Supplement: S1 Text — Supporting information including Appendices A and B and S1 and S2 Tables. (DOCX) [file pone.0239009.s002.docx]

***Supplementary Information***

**Building a green Belt and Road: a systematic review and comparative assessment of the Chinese and English-language literature**

Hoong Chen Teo ^1^, Ahimsa Campos-Arceiz ^1,2,3*^, Binbin Li ^4^, Mingquan Wu ^5^, Alex Mark Lechner ^1^

^1^ School of Environmental and Geographical Sciences, University of Nottingham Malaysia, Malaysia;

^2^ Southeast Asia Biodiversity Research Institute, Chinese Academy of Sciences, Myanmar

^3^ Center for Integrative Conservation, Xishuangbanna Tropical Botanical Garden, Chinese Academy of Sciences, China

^4^ Environmental Research Center, Duke Kunshan University, China;

^5^ State Key Laboratory of Remote Sensing Science, Aerospace Information Research Institute, Chinese Academy of Sciences, China;

* Corresponding author

## Appendix 1

Source: BRI environmental paper (Foggin, 2018). Average sentiment: 0.67

| Environmental conservation has developed significantly in China over the past 20 years, including more collaborative approaches and recent advances in establishing a national parks system. Community engagement and participation in developing localized plans for natural resource utilization and conservation have been critical features of successful ventures. Government programs and policies, the emergence of grassroots civil society, and the development of herders’ cooperatives and protected areas, are all tracked, each pointing towards the significant value of inclusive biodiversity conservation approaches for meeting broadly agreed development agendas, such as achieving the Sustainable Development Goals by 2030. |
| --- |

Source: BRI environmental paper (Yu et al., 2019). Average sentiment: -0.51

| Natural disasters like typhoons, floods and thunderstorms hit countries along the line frequently, and the economic losses caused by natural disasters to these areas are enormous. Carrying out research on natural disasters is of great significance for the government’s disaster prevention and mitigation, which can reduce disaster damage and social impact. For instance, China’s coastal areas have suffered lots of typhoon disasters that have resulted in considerable casualties and property damages. |
| --- |

Source: BRI news article (Ma and Yao, 2015). Average sentiment: 0.34

| The China-proposed "Belt and Road" initiatives reflect the common aspiration of countries along the routes and the implementation of the blueprint will bring huge benefits for Asia and the rest of the world. This modern version of the world-renowned ancient trade routes spans Asia, Africa and Europe, and each country along the routes has its own economic strength it wishes to share and also shortcomings that could only be addressed with the help from outside. With an eye to economic diversity in the region, the "Belt and Road" initiatives seek to promote win-win cooperation among participating nations by breaking the infrastructure bottlenecks, by boosting efficient allocation of resources and by further integration of markets. Aside from explaining how the development plan will bring about benefits for countries involved, a recently-released blueprint for the "Belt and Road" initiatives also outlines the principles, cooperation priorities and the mechanisms to facilitate progress. The rich content of the roadmap-style document testifies to China's sincerity in promoting common development in neighboring countries and also for partners on a different continent. The "Belt and Road" initiatives are like an invitation by China for countries to ride its express train of economic development. |
| --- |

Source: BRI news article (Greer, 2018). Average sentiment: -0.165

| In Bangladesh authorities recently blacklisted China Harbour Engineering Company, one of the region's most active BRI construction firms, on accusations of corruption. Burma was so alarmed by regional trends that it put a hold on its own BRI-funded port project in Kyaukpyu until the Chinese agreed to cut its scale by 80 percent. Nepal and Pakistan have also demanded that China cancel or completely retool ongoing projects in their countries. In western Pakistan opposition to the initiative has turned violent. Last week Baluchi separatists attacked the Chinese consulate in Karachi, treating Chinese infrastructure investment in their region as a threat to their dreams of independence. Chinese analysts who hoped that the BRI investment would help stabilize China's borderlands and ease the threat it faces from ethnic separatists inside China now must come to terms with an initiative that is embroiling China in conflict with separatists outside of it. The problems China has had with the BRI stem from contradictions inherent in the ends party leaders envision for the initiative and the means they have supplied to reach them. |
| --- |

## Appendix 2

Scopus database was queried on 15 Aug 2019 for BRI environmental papers between 1 Jan 2013 and 30 Jun 2019. A total of 262 results were returned, and after screening, 144 were deemed relevant. Search string:

(TITLE-ABS-KEY("green" AND "One Belt One Road" ) ) OR (TITLE-ABS-KEY("ecolog*" AND "One Belt One Road" ) ) OR (TITLE-ABS-KEY("environment*" AND "One Belt One Road" ) ) OR (TITLE-ABS-KEY("green" AND "Belt and Road Initiative" ) ) OR (TITLE-ABS-KEY("ecolog*" AND "Belt and Road Initiative" ) ) OR (TITLE-ABS-KEY("environment*" AND "Belt and Road Initiative" ) ) OR (TITLE-ABS-KEY("green" AND "Silk Road Economic Belt" ) ) OR (TITLE-ABS-KEY("ecolog*" AND "Silk Road Economic Belt" ) ) OR (TITLE-ABS-KEY("environment*" AND "Silk Road Economic Belt" ) ) OR (TITLE-ABS-KEY("green" AND "Maritime Silk Road" ) ) OR (TITLE-ABS-KEY("ecolog*" AND "Maritime Silk Road" ) ) OR (TITLE-ABS-KEY("environment*" AND "Maritime Silk Road" ) )

CNKI database was queried on 15 Aug 2019 for BRI environmental papers between 1 Jan 2013 and 30 Jun 2019. A total of 536 results were returned, and after screening, 297 were deemed relevant. Search string:

(KY='绿色'*'一带一路'+'一带一路'*'生态'+'一带一路'*'环境' OR AB='绿色'*'一带一路'+'一带一路'*'生态'+'一带一路'*'环境' OR KY='绿色'*'丝绸之路经济带'+'丝绸之路经济带'*'生态'+'丝绸之路经济带'*'环境' OR AB='绿色'*'丝绸之路经济带'+'丝绸之路经济带'*'生态'+'丝绸之路经济带'*'环境' OR KY='绿色'*'海上丝绸之路'+'海上丝绸之路'*'生态'+'海上丝绸之路'*'环境' OR AB='绿色'*'海上丝绸之路'+'海上丝绸之路'*'生态'+'海上丝绸之路'*'环境') AND (TI='绿色'*'一带一路'+'一带一路'*'生态'+'一带一路'*'环境'-'投资环境'-'论坛' OR TI='绿色'*'丝绸之路经济带'+'丝绸之路经济带'*'生态'+'丝绸之路经济带'*'环境'-'投资环境' -'论坛' OR TI='绿色'*'海上丝绸之路'+'海上丝绸之路'*'生态'+'海上丝绸之路'*'环境'-'投资环境' -'论坛' )

Scopus database was queried on 15 Aug 2019 to determine number of BRI papers between 1 Jan 2013 and 30 Jun 2019.

(TITLE-ABS-KEY("One Belt One Road" ) ) OR (TITLE-ABS-KEY("Belt and Road Initiative" ) ) OR (TITLE-ABS-KEY("Silk Road Economic Belt" ) ) OR (TITLE-ABS-KEY("Maritime Silk Road" ) ) AND (DOCTYPE (“ar”) )

CNKI database was queried on 15 Aug 2019 to determine number of BRI papers between 1 Jan 2013 and 30 Jun 2019.

(KY='一带一路' OR AB='一带一路' OR KY='丝绸之路经济带' OR AB='丝绸之路经济带' OR KY='海上丝绸之路' OR AB='海上丝绸之路')

Scopus database was queried on 15 Aug 2019 for oil palm environmental papers between 1 Jan 2013 and 30 Jun 2019. A total of 262 results were returned, and after screening, 144 were deemed relevant. Search string:

( TITLE-ABS-KEY ( "oil palm" AND "environmental impact*" ) ) OR ( TITLE-ABS-KEY ( "oil palm" AND "sustainab*" ) ) AND ( LIMIT-TO ( PUBYEAR , 2019 ) OR LIMIT-TO ( PUBYEAR , 2018 ) OR LIMIT-TO ( PUBYEAR , 2017 ) OR LIMIT-TO ( PUBYEAR , 2016 ) OR LIMIT-TO ( PUBYEAR , 2015 ) OR LIMIT-TO ( PUBYEAR , 2014 ) ) AND ( LIMIT-TO ( DOCTYPE , "ar" ) ) AND ( LIMIT-TO ( SRCTYPE , "j" ) )

Factiva was queried on 29 Jul 2020 for BRI environmental news articles between 1 May 2019 and 30 Jun 2019. 141 English-language results and 210 Chinese-language results were obtained. Search strings:

(一带一路 and 生态) or (一带一路 and 生态环境)

"Belt and Road" and environment*

Factiva was queried on 29 Jul 2020 for oil palm environmental news articles between 1 Jan 2019 and 30 Jun 2019. 239 results were obtained. Search strings:

"oil palm" and environment*

## Supplementary Table 1. Institutional affiliations of Chinese first authors for both Chinese and English language papers.

| **Institution type** | **Number of papers** |
| --- | --- |
| Chinese Academy of Sciences | 24 |
| Communist Party schools | 8 |
| Project 211 universities | 147 |
| Other universities | 138 |
| Colleges | 62 |
| Research centres | 8 |
| Corporations | 9 |
| Other organisations | 11 |

## Supplementary Table 2. Summary of themes in “The Belt and Road Ecological and Environmental Cooperation Plan”.

| **The Belt and Road Ecological and Environmental Cooperation Plan (official translation)** | **“一带一路”生态环境保护合作规划** | **Summary** | **Themes** |
| --- | --- | --- | --- |
| I. Significance | 一、重要意义 | Rationale and significance of environmental protection in BRI; principles and goals of a green BRI | Principles |
| II. Overarching Requirements | 二、总体要求 |  |  |
| III. Highlight the Concept of Ecological Civilization and Strengthen Policy Coordination | 三、突出生态文明理念，加强生态环保政策沟通 | Green policy coordination, with an emphasis on ecological civilisation (including green accounting) | Policy |
| IV. Respect Laws and Regulations to Promote International Production Capacity Cooperation and Eco-friendly Infrastructure Construction | 四、遵守法律法规，促进国际产能合作与基础设施建设的绿色化 | Use laws and regulations to promote green production and infrastructure | Law |
| V. Promote Sustainable Production and Consumption and Boost Green Trade | 五、推动可持续生产与消费，发展绿色贸易 | Promote sustainable production, consumption and trade | Trade |
| VI. Increase Support for Green Financing to Boost Financial Integration | 六、加大支撑力度，推动绿色资金融通 . | Green financing | Finance |
| VII. Carry out Eco-Environmental Protection Projects and Activities to Enhance People-to-People Bonds | 七、开展生态环保项目和活动，促进民心相通 | Develop environmental protection programmes to promote people-to-people bonds | Protection |
| VIII. Step up Capacity Building and Make Use of Local Advantages | 八、加强能力建设，发挥地方优势 | Step up capacity building, making use of local advantages, measures to safeguard success of green BRI | Capacity |
| IX. Major Projects | 九、重大项目 |  |  |
| X. Safeguard Measures | 十、保障措施 |  |  |
